# Role of High-Resolution Computed Tomography in Double-Lumen Tube Selection for Patients Undergoing Minimally Invasive Coronary Bypass

**DOI:** 10.3390/jcm15145415

**Published:** 2026-07-10

**Authors:** Mesher Ensarioğlu, Alperen Kutay Yıldırım

**Affiliations:** 1 Department of Anesthesiology and Reanimation, Gulhane Training and Research Hospital, University of Health Sciences, Ankara 06010, Turkey; 2 Department of Cardiovascular Surgery, Gulhane Training and Research Hospital, University of Health Sciences, Ankara 06010, Turkey; drayildirim@yahoo.com

**Keywords:** double-lumen tube, one-lung ventilation, high-resolution computed tomography, airway measurement, minimally invasive coronary artery bypass, lung isolation

## Abstract

**Background:** No consensus guideline exists for double-lumen tube (DLT) size selection, which conventionally relies on height, sex, and chest radiography. Computed tomography (CT) has been proposed to refine sizing, particularly in women and patients of small stature. We examined whether high-resolution CT (HRCT) measurement of the trachea and main bronchi improves DLT size selection beyond a height- and sex-based estimate in patients undergoing minimally invasive coronary artery bypass (MICS-CABG). The aim of this study was to determine whether HRCT measurement of the trachea and main bronchi improves DLT size selection beyond a height- and sex-based estimate in patients undergoing MICS-CABG. **Methods:** A total of 140 patients were retrospectively analyzed who had undergone MICS-CABG and had HRCT within 30 days of surgery. Tracheal anteroposterior and transverse diameters and left and right main bronchus diameters were measured in lung, mediastinal, and bronchial windows (mean of six readings by two observers). The DLT size used was related to each parameter through Spearman correlation; the agreement between the height/sex estimate and the size used was determined using Cohen’s kappa; discrimination for a larger tube (≥39 Fr) was evaluated through receiver operating characteristic analysis; and the incremental value of imaging was assessed using leave-one-out cross-validation. **Results:** In total, 82.1% of patients were male (mean age 59.7 ± 10 years; height 171.7 ± 7.3 cm). The height- and sex-based estimates correlated most strongly with the size used (ρ = 0.885, *p* < 0.001), followed by height alone (ρ = 0.844, *p* < 0.001); the best imaging measure, the lung-window left main bronchus diameter, was weaker (ρ = 0.503, *p* < 0.001), and body mass index showed no association (ρ = −0.008, *p* = 0.926). The estimate matched the size used exactly in 85.7% of patients and was within one size for all patients (linear-weighted κ = 0.831). Height best discriminated a larger tube (AUC = 0.969). Adding the bronchial diameter to height and sex did not improve cross-validated prediction (exact match 80.7% with and without; AUC 0.972 versus 0.973). The mediastinal window yielded systematically larger calibers than the lung and bronchial windows. **Conclusions:** HRCT airway measurement did not improve the prediction of the double-lumen tube size selected in routine practice beyond a simple height- and sex-based formula. Because the reference standard was the size clinicians actually placed rather than an independently validated optimum, these data argue against routine preoperative HRCT undertaken solely to predict tube size; they do not exclude a benefit of HRCT when selection is judged against clinical outcomes. CT remains valuable when abnormal airway anatomy is suspected.

## 1. Introduction

High-resolution computed tomography (HRCT) is a type of computed tomography designed to achieve higher image resolution. It is primarily used to assess the lung parenchyma and is especially preferred for interstitial lung diseases. For the evaluation of a difficult airway with either a single-lumen or a double-lumen tube (DLT), tomographic imaging is not routinely performed. Demographic characteristics, including height and weight, along with findings on direct chest radiography, have been used to estimate overall DLT size. However, for patients undergoing preoperative evaluation with known comorbidities, such as those undergoing cardiovascular surgery, additional imaging is often required, including tomographic imaging. Studies suggest that radiographic differences may be observed during these evaluations. Muro et al.’s study highlights a difference in tracheal size between patients with chronic obstructive pulmonary disease (COPD) and the general population [1].

Tomographic evaluation of tracheal size, compared with traditional estimation methods, is an area of interest in the literature. Williamson et al.’s review, published by the European Respiratory Society, states that tomographic evaluation of the trachea and distal airways is a reliable method for patients with known respiratory comorbidities [2]. This review also supports the reliability of three-dimensional airway reconstruction and other imaging-based reconstructions. Suvari et al., in their study of 55 patients, used tomographic imaging for DLT. By selecting a tube 0.5 to 1 mm below the tomographically calculated diameter, successful lung separation was achieved in 92.7% of patients. Compared with routine evaluation, in which 54.9% of patients required a tube size change, tomographic findings were found adequate. Additional statements recommend combining height, weight, age, and chest radiography findings [3].

Several methods exist for tomographic distance calculations, including that of Suvvari et al., who used axial reconstruction and measured tracheal circumference 5 cm above the carinal base and relevant bronchial structure circumferences 1 to 2 mm above the carinas [3]. Mihatsch et al.’s study focused on sex discrepancies in tomographic calculations and reported that results from three 3D reconstructions were more reliable, especially for female patients [4]. Different windows for evaluation were also studied, with Seo et al. reporting that bronchial windows (Hounsfield unit, HU, between 1000 and −450) were more reliable than both lung windows (between 1500 HU and −700 HU) and mediastinal windows (between 400 HU and 25 HU). These available studies in the literature suggest that a more extensive approach may be warranted regarding which modality should be used from available tomographic slides for the estimation of DLT size [5].

Combinations of distances were used in the study by Aljathlany et al., which was a retrospective study of 100 patients. In this study, in addition to the tracheal posteroanterior distance and transverse diameter, the cross-sectional area of the trachea was also evaluated, with separate calculations performed at the glottic, proximal, and subglottic levels [6]. Overall, it was stated that routinely utilized parameters, including age, gender, and neck circumference, still played a statistically significant role in regression analysis.

Minimally invasive coronary artery bypass is increasingly performed as an alternative to median sternotomy in selected patients, and current revascularization guidelines recognize minimally invasive surgical approaches within the management of coronary artery disease [7,8]. Performed through a small thoracotomy, these procedures depend on one-lung ventilation to provide surgical access and a still operative field, so that reliable lung isolation is a prerequisite, and appropriate tube sizing is correspondingly relevant in this population.

Lung isolation for one-lung ventilation can be achieved with a double-lumen tube or a bronchial blocker, and the double-lumen tube remains the most widely used device for most thoracic and an increasing number of minimally invasive cardiac procedures [9,10,11]. Selecting an appropriately sized tube is clinically important: an oversized tube risks bronchial laceration, mucosal ischaemia, postoperative sore throat and hoarseness, whereas an undersized tube predisposes to incomplete lung collapse, gas leak around the bronchial cuff and intraoperative malposition [12,13,14]. Bronchial blockers provide an alternative when airway anatomy is unfavorable or a single-lumen tube is already in situ, and several trials report fewer airway symptoms and possibly fewer postoperative pulmonary complications with blockers, albeit with slower or less complete lung collapse and more frequent intraoperative repositioning [15,16,17,18]. Because no consensus guideline for double-lumen tube size selection exists, a range of approaches has been proposed, extending from demographic rules and chest-radiographic tracheal width to cross-sectional measurement of the trachea, cricoid cartilage and main bronchi on computed tomography, ultrasonographic measurement of the cricoid, three-dimensional airway reconstruction or printing, and machine learning models trained on radiographic and demographic inputs [19,20,21,22,23,24,25]. Several of these reports suggest that augmenting demographic information with an imaging measure improves accuracy, most notably among women and patients of small stature, in whom demographic rules perform least well; whether such a combination adds value across a broader surgical population, however, remains unresolved [26].

In this study, the combination of different tomographic windows and localizations for distance measurements was evaluated to estimate the potential predictive roles of DLT size and the need to change tube size in patients undergoing minimally invasive coronary bypass surgery. The study hypothesized that different tomographic windows at different segments may provide additional insight into tube selection.

## 2. Materials and Methods

The study was conducted as a single-center retrospective study at a tertiary training and research hospital, with a focus on trauma and cardiovascular surgery. An initial investigation of the study’s suitability and data was conducted by the hospital’s committee for medical studies. After approval from the university’s ethics committee, the “Gülhane Scientific Research Ethics Committee,” with approval date 12 February 2026 and approval number 2026/32, patient evaluation began. Patients who had undergone intubation with a double-lumen tube for minimally invasive coronary artery bypass (MICS-CABG) surgery were included in the study population, and were evaluated retrospectively beginning on 1 January 2026, and extending back until the required patient count was reached.

The inclusion criteria for the study were being over 18 years old, requiring intubation with a DLT, and having an HRCT performed within thirty days prior to the operation. Exclusion criteria were a history of head-and-neck or airway surgery, known tracheal stenosis or congenital tracheobronchial malformation, an endoluminal or extrinsic airway-obstructing mass, the absence of a high-resolution CT within 30 days of surgery, and the use of a right-sided or single-lumen tube or a tube size outside the 35–41 French range. Demographic characteristics of patients (age, gender, height and weight), presence of comorbidities, chest radiography and HRCT imaging were recorded. For tomographic evaluation, lung (between 1500 HU and −700 HU), mediastinal (between 400 HU and 25 HU) and bronchial (between 1000 HU and −450 HU) windows were used to calculate distances. In each window, the tracheal anteroposterior and transverse distances and the diameters of the left and right main bronchi were recorded.

Tracheal distances were measured 5 cm above the carina, while the main bronchial diameters were measured 1 to 2 mm distal to the carina (below the bifurcation). All calculations were performed three times at each window by two different authors and were accepted if the estimated difference between each author’s calculations was less than 5%. Afterward, the exact value would be given as the mean of all six estimations.

Double-lumen tube size was selected prospectively by the attending anesthesiologist during routine clinical care, on the basis of the patient’s sex and height alone, and before intubation; the anesthesiologist did not have access to the computed-tomographic airway measurements. In men, a 37 French tube was chosen below 170 cm, 39 French from 170 to 179 cm, and 41 French at 180 cm or above; in women, a 35 French tube was chosen below 160 cm, and 37 French was used at 160 cm or above. This sex- and height-based selection constitutes the “clinical estimate” definition. All patients were intubated with a left-sided double-lumen tube; no right-sided tubes were used. All tubes were from a single manufacturer (Tuoren double-lumen endobronchial tube; Tuoren Medical, Changyuan, Henan, China). After induction, tube position was confirmed in every case using flexible fiberoptic bronchoscopy: through the tracheal lumen, the carina was identified and the bronchial limb was confirmed within the left main bronchus, with the proximal bronchial cuff seated just distal to the carina. The selected size was accepted when the tube advanced into the left main bronchus without undue resistance; the bronchial cuff achieved an effective seal at a low inflation volume (approximately 2–3 mL) with satisfactory collapse of the non-dependent lung and no audible leak. The size was changed when these conditions were not met: a smaller tube was substituted when passage met resistance or bronchoscopy suggested mucosal compression, and a larger tube was used when the bronchial cuff required a substantially greater volume to seal, a leak persisted around the cuff, lung collapse was incomplete, or the tube migrated proximally.

All parameters were initially recorded in a Microsoft Office 365 Excel spreadsheet without personal identification. After an initial assessment for missing values, IBM SPSS Statistics Premium Gradpack 30 Edition was used for statistical analysis. Parameters were evaluated for distribution using histograms and the Kolmogorov–Smirnov test. Based on the distributions, parameters were reported either as a mean and standard deviation or as a median and 25th to 75th percentiles.

Correlations between demographic, radiographic and tomographic parameters and the double-lumen tube size used were assessed using the Spearman rank correlation coefficient. Agreement between the height- and sex-based size estimate and the size actually used was quantified with Cohen’s kappa (unweighted and linear-weighted). The ability of each parameter to discriminate the use of a larger tube was evaluated using receiver operating characteristic (ROC) analysis; the area under the curve (AUC) was reported, and optimal cut-off values were identified using the Youden index. A two-tailed *p* value < 0.05 was considered statistically significant.

Regarding the study size, after a literature review, an estimated range of 80 to 100 patients was assumed. For a reliable statistical analysis to detect a statistical difference between two groups on a scale level with 80% power and a 5% type I error, at least 128 patients were required. For a possible regression analysis with three predictor parameters, assuming the same power and type I error rate, at least 126 patients were required to detect a small effect (0.2) of one parameter. Considering 10% inadequate data and the inadequacy of additional parameters, including comorbidities and imaging modalities, for evaluation, 140 patients were to be recruited in the study.

## 3. Results

**Figure 1:**
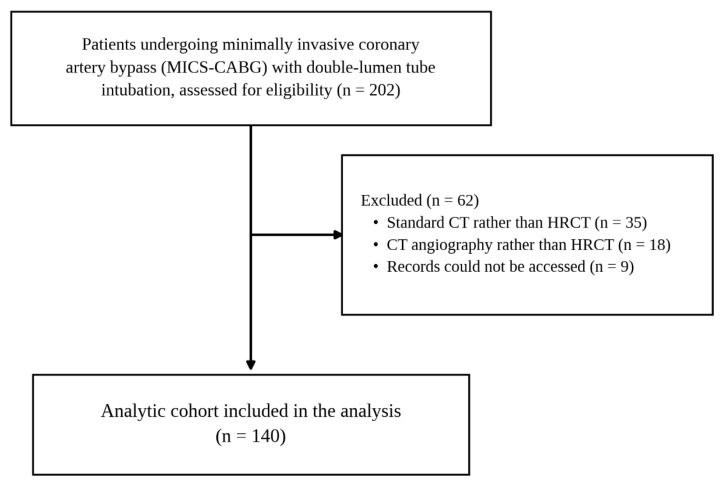
Flow chart of patient selection.

A total of 202 patients were screened; 62 were excluded (35 had standard computed tomography rather than HRCT, 18 had computed tomography angiography, and nine had records that could not be accessed), leaving 140 patients in the analytic cohort (Figure 1). The cohort was predominantly male (82.1%), with a mean age of 59.7 (±10) years and a mean height of 171.7 (±7.3) cm. Hypertension (*n* = 91, 65%), diabetes mellitus (*n* = 65, 46.4%), and hyperlipidemia (*n* = 65, 46.4%) were the most frequent comorbidities (Table 1).

Across the lung, mediastinal, and bronchial windows, respectively, the tracheal anteroposterior diameter was 1.79 (±0.32), 2.03 (±0.32), and 1.81 (±0.31) cm, and the tracheal transverse diameter was 1.91 (±0.32), 2.19 (±0.37), and 1.95 (±0.32) cm. The left main bronchus measured 1.00 (±0.18), 1.21 (±0.20), and 1.06 (±0.24) cm, and the right main bronchus was 0.84 (±0.21), 1.05 (±0.23), and 0.88 (±0.26) cm across the same three windows. On chest radiography, the tracheal transverse diameter was 1.89 (±0.28) cm. For every tracheal and bronchial structure, the mediastinal window yielded systematically larger values than the lung and bronchial windows. The three windows were strongly correlated with one another for each structure (Pearson r 0.68–0.95 (Table 2)).

The size of the double-lumen tube actually used was 35 Fr in nine patients, 37 Fr in 29, 39 Fr in 73 and 41 Fr in 29. The height- and sex-based clinical estimate showed the strongest association with the size used (ρ = +0.885, *p* = 0.001), followed by height alone (ρ = +0.844). Among the imaging measurements, the lung-window left main bronchus diameter had the strongest correlation (ρ = +0.503, *p* = 0.001). Body mass index was not associated with tube size (ρ = −0.008, *p* = 0.926) (Table 3).

Agreement between the height- and sex-based estimates and the size used was high, with an exact match in 120 (85.7%) patients, and in every patient they agreed to within one tube size (linear-weighted κ = 0.831). A tube size different from the clinical estimate was used in 20/140 (14.3%) patients, with no systematic direction (12 larger and 8 smaller than predicted). In the receiver operating characteristic analysis, height had the highest discrimination for the requirement of a larger tube (≥39 Fr) (AUC = 0.969). The best-performing imaging parameter, the lung-window left main bronchus diameter, achieved an AUC of 0.800 at an optimal cut-off of 0.90 cm (Table 4).

To test whether tomographic measurement adds to demographic information, candidate models predicting the size used were compared through leave-one-out cross-validation. Adding the lung-window left main bronchus diameter to the height and sex did not improve prediction (exact match 80.7% with and without the measurement; cross-validated AUC for a larger tube 0.972 versus 0.973), and adding weight conferred no further benefit. The height- and sex-based clinical estimate alone remained the single best predictor (exact match 85.7%, cross-validated R^2^ 0.787) (Table 5).

**Figure 2:**
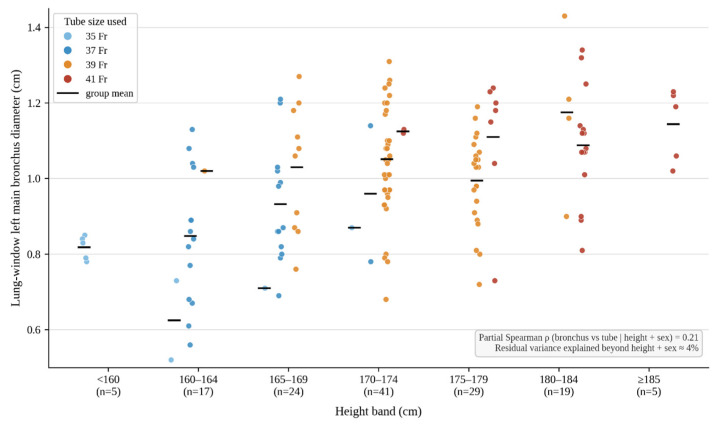
Lung-window left main bronchus diameter by tube size used.

As height was the dominant determinant of the size used, patients were grouped into 5 cm height bands to assess the contribution of the lung-window left main bronchus diameter while holding stature approximately constant (Figure 2). Within bands containing more than one tube size, patients who received a larger tube tended to have a slightly larger bronchial diameter. This residual association was weak: the partial Spearman correlation between bronchial diameter and tube size was 0.31 after adjustment for height and 0.21 after adjustment for height and sex. Within height strata, therefore, the tomographic measurement did not separate the tube sizes used at the level of the individual patient (Supplementary Table S1).

Because double-lumen tube size is an ordered categorical variable, a proportional-odds ordinal logistic model of size as a function of height and sex was also fitted. Height was strongly associated with a larger tube (odds ratio 1.68 per cm, 95% CI 1.47–1.92; 13.3 per 5 cm), as was male sex (odds ratio 18.2, 95% CI 1.8–182.6). The model assigned 82.9% of patients to the exact size used and placed all patients within one size. To test whether dichotomizing at ≥39 Fr obscured the resolution of adjacent sizes, discrimination was assessed at each adjacent boundary. Height separated adjacent sizes well, including the densest boundary of 37 versus 39 Fr (AUC 0.954, 95% CI 0.914–0.995), whereas the best imaging measure was weaker at every boundary (Supplementary Table S2).

Each point represents one patient; horizontal bars denote group means. Within most bands, the mean bronchial diameter increases with tube size, but distributions for adjacent sizes overlap substantially. The partial Spearman correlation between bronchial diameter and tube size, adjusting for height and sex, was 0.21.

## 4. Discussion

In this retrospective cohort of 140 patients undergoing minimally invasive coronary artery bypass, the question of whether HRCT measurements of the trachea and main bronchi across three window settings could improve DLT size selection beyond the conventional height- and sex-based estimate was investigated. The height/sex estimate was the strongest determinant of the size actually used. Among the imaging measurements, the left main bronchus diameter in the lung window was the strongest single correlate of tube size. Across all assessments, the three computed tomography windows ranked patients consistently, yet the mediastinal window yielded larger absolute calibers than the lung and bronchial windows. Adding a tomographic measurement to height and sex did not improve cross-validated prediction of the size used.

The strong performance of the height- and sex-based estimate is consistent with current evidence on demographic DLT selection. Brodsky and colleagues reported that an anthropometric assessment, derived from tracheal width and, by extension, patient stature, predicts an appropriate left DLT in most patients, and that height and sex remain the default in routine practice [13,19,27]. In our cohort, this approach agreed with the size ultimately used for the majority of patients, with no systematic tendency toward larger or smaller tubes. This contrasts with reports that conventional demographic methods overestimate the adequate size in individual patients on three-dimensional reconstruction and that a substantial proportion of patients require a tube change when sized conventionally [3,4]. Part of this discrepancy is due to the reference standard, as the study benchmarked predictors against the size clinicians actually placed rather than against an independently defined anatomical optimum. Thus, the high agreement indicates that practice in the unit mostly used height and sex.

Supplementing demographic data with an airway measurement might have refined selection; according to the available literature, it was tested using leave-one-out cross-validation. Adding the lung-window left main bronchus diameter to height and sex did not change the exact-match prediction of the size used and barely altered discrimination for a larger tube (area under the curve 0.972 rising to 0.973); adding weight contributed nothing further, and the height/sex estimate alone remained the single best predictor. The apparent advantage of combining imaging with clinical parameters seen in smaller series, including the combination of cricoid ring and left bronchial diameters, tracheal ultrasound, and computed-tomographic bronchial measurement, is therefore likely to reflect the small samples [26,28,29,30]. Once height and sex are known, a single airway diameter provides little independent information about the eventual size chosen. These findings limit the role of performing preoperative HRCT solely to size the DLT. This was further confirmed when patients were considered within narrow height strata: although larger bronchi were intubated with larger tubes at any given stature, the residual association was weak and was attenuated after accounting for sex, indicating that much of this within-stratum signal reflects sex differences in bronchial caliber rather than independent anatomical information. Together with the overlap in bronchial diameter between adjacent tube sizes, this explains why a tomographic measurement did not improve size selection even within height groups. The minority of patients in whom the placed tube differed from the demographic estimate might have been expected to be those in whom airway imaging adds most. However, these patients were demographically and anatomically indistinguishable from the remainder, and the direction of deviation was unrelated to bronchial caliber, so computed tomography would not have predicted the overrides.

The study’s findings fall within the existing literature on imaging and model-based sizing. Beyond the single bronchial measurements, investigators have matched the double-lumen tube cuff to the airway diameter measured on computed tomography, paired computed-tomographic measurement with video-bronchoscopic guidance to place right-sided tubes, selected tubes from ultrasonographic cricoid measurement, and reconstructed or three-dimensionally printed the airway to choose a size, while machine learning models trained on radiographic and demographic inputs have been utilized for prediction [21,22,24,31,32]. A recent systematic review concluded that no single parameter has proven definitive [19]. The recurring message, which the study’s cross-validation reinforces, is that imaging refines selection chiefly at the extremes, while for the average patient demographic, estimation performs comparably, and similar imaging-based studies have been reported for endotracheal tube sizing in other populations [25,33]. A distinction in the reference standard helps reconcile these results with the wider literature. Many imaging-based studies benchmark tube selection against an independent anatomical target such as the three-dimensional airway reconstruction of Mihatsch and colleagues, against which conventional demographic methods overestimated the adequate size, or the morphometric reference values of Min and colleagues [4,25]. Against such anatomical or functional standards, airway measurement can demonstrate value that a demographic rule does not capture. The present study, by contrast, benchmarks predictors against the size clinicians selected, which was itself guided largely by height and sex. A similar observation was reported by Smeltz and colleagues, relating computed-tomographic left bronchial diameter to the selected size, which supports our finding that this relationship exists but is weaker than that with stature [23]. When the reference standard is the size chosen in routine practice rather than an anatomical optimum or a clinical outcome, height and sex are difficult to improve upon, and high-resolution computed tomography accordingly appears less useful here than in studies designed around anatomical fit or airway complications. This, however, does not exclude the benefit of imaging when judged against those endpoints.

More broadly, the principle that an imaging marker should be validated against clinically meaningful endpoints before it enters routine decision-making recurs throughout cardiovascular procedural imaging, with examples ranging from intracardiac echocardiographic guidance, whose adoption in atrial fibrillation ablation has been weighed against procedural complications and efficacy, to multimodal atrial substrate imaging, whose thresholds await validation before clinical use [34,35]. Our findings sit within this frame: a tracheobronchial dimension that correlates with the selected size is not, by itself, an established tool for improving selection and is a marker anchored to clinical practice rather than to outcomes.

Among the imaging measurements, the lung-window left main bronchus diameter had the strongest observed correlation with the size used. This was a predicted observation, as the bronchial limb of a left-sided DLT is seated in the left main bronchus. The left bronchial diameter is the dimension used for computed-tomographic and radiographic selection in the studies of Hannallah and Brodsky [27,28]. Nonetheless, the strength of this association was modest and inferior to that of height, indicating that a single bronchial diameter is not superior to overall stature in this population.

The three computed tomography windows were highly correlated for every airway structure, but they were not interchangeable, as the mediastinal window yielded systematically larger diameters, with the left main bronchus measuring 1.00 cm on the lung window versus 1.21 cm on the mediastinal window. Seo and colleagues similarly demonstrated that the chosen window materially affects bronchial measurement and proposed a specific setting to guide DLT selection [5]. The practical implication is that any fixed computed-tomographic diameter threshold for sizing must specify the window used, because the same bronchus measured using the mediastinal window can read as close to a French size larger compared to measurements using the lung or bronchial window, which could bias selection toward an oversized tube.

Height and sex dominated the prediction, whereas the body mass index showed no association with the size used. This is consistent with body stature, rather than adiposity, governing airway caliber, and complements the multivariable airway-imaging selection tool of Aljathlany and colleagues, in which age, sex, and neck circumference, but not measures of body fat, were observed as significant predictors [6]. Together, these observations support the statement that the determinants of DLT size are skeletal and sex-related.

The clinical importance of precise double-lumen tube sizing also depends on the chosen isolation strategy. When a bronchial blocker is used, tube-size selection is effectively replaced by blocker positioning. Randomized and cohort data suggest that blockers produce fewer airway symptoms and may be associated with fewer postoperative pulmonary complications, at the cost of slower or less complete lung collapse and more frequent intraoperative repositioning [15,16,17,18]. In patients with a difficult airway, lung isolation may be achieved with a single-lumen tube and a blocker rather than a double-lumen tube [12]. These alternatives do not diminish the importance of correct sizing once a double-lumen tube is selected, but they place it in context: the marginal benefit of obtaining preoperative computed tomography solely to size a tube must be weighed against equally effective isolation strategies that require no such imaging. The implications of these findings for the postoperative period are indirect but real. In minimally invasive coronary surgery, the adequacy of lung isolation governs surgical exposure, while tube-related airway trauma, sore throat, and hoarseness contribute to early postoperative morbidity. Our results indicate that, for selecting tube size, a height- and sex-based estimate is sufficient for most patients, thereby avoiding both an oversized tube (risking mucosal injury) and an undersized tube (risking incomplete collapse) without routine preoperative tomography. Airway management of this kind contributes to a smooth perioperative course rather than to longer-term cardiovascular prognosis, which in coronary artery disease is governed mainly by guideline-directed therapy and structured rehabilitation [36].

The reported findings should be read with caution, as the analysis was anchored to the size clinicians selected rather than to clinical outcomes; it cannot establish whether more accurate sizing, by any method, alters patient-centered endpoints. The prognostic relevance of correct sizing most plausibly lies in avoiding airway injury and the hypoxemia of incomplete isolation, but our data cannot quantify this. Future work should relate preoperative airway measurement, ideally including three-dimensional reconstruction, to prospectively collected endpoints such as first-attempt placement, tube exchange, quality of lung collapse, audible cuff leak, and postoperative sore throat, hoarseness, and airway trauma.

This study has several limitations, the most important of which concerns the reference standard. The outcome was the tube size clinicians actually placed, a choice itself largely guided by height and sex; the strong performance of the height- and sex-based estimate is therefore in part circular, and our analysis tests how well measurements predict local clinical practice rather than an independently validated optimal size or a clinically meaningful endpoint. We did not have reliable data on outcomes that would define potential further roles of CT, such as first-attempt placement success, the need for tube exchange, malposition, audible cuff leak, the quality of lung collapse, airway-pressure abnormalities, postoperative sore throat or hoarseness, and airway trauma. Therefore, it could not be stated whether HRCT measurement would improve selection when judged against these endpoints rather than against the size chosen in practice; a prospective study relating preoperative airway measurement to these intraoperative and postoperative outcomes would be needed to resolve this. Another limitation was that measurements were two-dimensional axial diameters rather than three-dimensional reconstructions, which may better capture the elliptical bronchial lumen. Beyond caliber, airway features such as the angulation of the proximal trachea and the glottic axis influence the ease of tube placement and were not assessed here [37]. The cohort was predominantly male and confined to minimally invasive coronary bypass with tube sizes between 35 and 41 French, which limits generalizability to other populations and to smaller tubes. A further limitation concerns measurement reproducibility. Although every airway dimension was measured independently by two observers and accepted only when their readings agreed within 5% before averaging, the individual observer values were not retained; formal inter- and intra-observer reliability could therefore not be quantified using intraclass correlation or Bland–Altman analysis. The agreement we report is consequently between computed-tomographic window settings rather than between observers, and a prospective study with retained observer-level readings would be required to confirm the reproducibility of the measurement technique.

## 5. Conclusions

Among patients undergoing minimally invasive coronary bypass, a simple estimate based on height and sex reliably predicted the double-lumen tube size selected in routine practice, and HRCT airway measurement did not improve on it. As the outcome modeled was the size clinicians actually placed rather than an independently validated clinical endpoint, these findings concern the prediction of local practice rather than anatomical optimality, and they do not support routine preoperative HRCT undertaken solely to predict tube size. They do not, however, exclude a role for HRCT when selection is judged against intraoperative and postoperative outcomes, which would require prospective study to test. Computed tomography retains value when airway anatomy is known or suspected to be abnormal, and if computed-tomographic measurements are used for sizing, the window setting should be standardized, given the systematic differences in caliber between windows.

## Figures and Tables

**Table 1:** Baseline demographic and clinical characteristics.

Demographic Characteristics		Mean (SD)
Age (years)		59.7 (10.0)
Gender (*n*, %)	Male	115 (82.1)
	Female	25 (17.9)
Height (cm)		171.7 (7.3)
Weight (kg)		80.8 (11.0)
BMI (kg/m^2^)		27.4 (3.0)
Smoking history	Ever-smoker (*n*, %)	105 (75.0)
	Pack-years (median, IQR)	30 (20–45)
**Comorbidities**		***n* (%)**
Hypertension		91 (65.0)
Diabetes mellitus		65 (46.4)
Hyperlipidaemia		65 (46.4)
Coronary artery disease		17 (12.1)
Benign prostatic hyperplasia		11 (7.9)
Hypothyroidism		8 (5.7)
Chronic obstructive pulmonary disease		4 (2.9)
Asthma		3 (2.1)
Obstructive sleep apnoea		2 (1.4)
Peripheral artery disease		3 (2.1)
Cerebrovascular disease		3 (2.1)
Gout		3 (2.1)

SD: standard deviation; IQR: Interquartile range; BMI: body mass index.

**Table 2:** Airway dimensions and inter-window agreement.

Airway Dimensions by Window (cm, Mean ± SD)				
**Structure**	**Chest X-ray**	**Lung window**	**Mediastinal window**	**Bronchial window**
Tracheal AP	—	1.79 ± 0.32	2.03 ± 0.32	1.81 ± 0.31
Tracheal transverse	1.89 ± 0.28	1.91 ± 0.32	2.19 ± 0.37	1.95 ± 0.32
Left main bronchus	—	1.00 ± 0.18	1.21 ± 0.20	1.06 ± 0.24
Right main bronchus	—	0.84 ± 0.21	1.05 ± 0.23	0.88 ± 0.26
**Inter-window agreement**				
**Structure**	**Window pair**	**Bias (cm)**	**95% limits of agreement (cm)**	**ICC**
Tracheal AP	Lung–Mediastinal	−0.234	−0.429 to −0.039	0.755
	Lung–Bronchial	−0.014	−0.266 to 0.239	0.918
	Mediastinal–Bronchial	0.220	−0.056 to 0.496	0.729
Tracheal transverse	Lung–Mediastinal	−0.284	−0.741 to 0.174	0.577
	Lung–Bronchial	−0.045	−0.451 to 0.361	0.786
	Mediastinal–Bronchial	0.239	−0.143 to 0.621	0.680
Left main bronchus	Lung–Mediastinal	−0.207	−0.419 to 0.004	0.522
	Lung–Bronchial	−0.051	−0.341 to 0.238	0.740
	Mediastinal–Bronchial	0.156	−0.178 to 0.490	0.562
Right main bronchus	Lung–Mediastinal	−0.207	−0.528 to 0.113	0.505
	Lung–Bronchial	−0.043	−0.425 to 0.339	0.655
	Mediastinal–Bronchial	0.164	−0.202 to 0.531	0.592

ICC, intraclass correlation coefficient (two-way random-effects model, absolute agreement, single measures); LoA, limits of agreement (bias ± 1.96 × SD of within-patient differences; Bland–Altman); bias is the first window minus the second, with a positive value indicating the first window reads larger. *n* = 140. HU thresholds: lung −700 to 1500; mediastinal 25 to 400; bronchial −450 to 1000. SD, standard deviation; AP, anteroposterior.

**Table 3:** Correlation of parameters with the double-lumen tube size used.

Parameter	Spearman ρ (95% CI)	*p* Value
**Clinical estimate (height/sex)**	**0.885 (0.843–0.916)**	<0.001
**Height**	**0.844 (0.788–0.886)**	<0.001
**Weight**	**0.503 (0.367–0.617)**	<0.001
Body mass index	−0.008 (−0.174–0.158)	0.926
Age	−0.207 (−0.366–−0.041)	0.014
Chest X-ray tracheal transverse	0.265 (0.104–0.413)	0.002
Lung—tracheal AP	0.411 (0.263–0.540)	<0.001
Lung—tracheal transverse	0.437 (0.292–0.562)	<0.001
**Lung—left bronchus**	**0.503 (0.368–0.618)**	<0.001
Lung—right bronchus	0.299 (0.140–0.443)	<0.001
Mediastinal—tracheal AP	0.444 (0.300–0.568)	<0.001
Mediastinal—tracheal transverse	0.397 (0.247–0.528)	<0.001
Mediastinal—left bronchus	0.418 (0.271–0.546)	<0.001
Mediastinal—right bronchus	0.256 (0.094–0.405)	0.002
Bronchial—tracheal AP	0.444 (0.300–0.568)	<0.001
Bronchial—tracheal transverse	0.384 (0.233–0.517)	<0.001
Bronchial—left bronchus	0.402(0.253–0.532)	<0.001
Bronchial—right bronchus	0.258 (0.097–0.407)	0.002

Bold rows denote ρ ≥ 0.50. CI: Confidence Interval.

**Table 4:** Agreement of tube size estimation and discrimination of a larger tube.

Clinical Estimation vs. the Size Used	Value (95% CI)			
Exact agreement	120/140 (85.7%)			
Linear-weighted κ	0.831 (0.755–0.899)			
Unweighted κ	0.778 (0.676–0.866)			
**ROC discrimination of tube size ≥ 39 Fr**				
**Predictor Factors**	**AUC (95% CI)**	**Youden Cut-off**	**Sensitivity**	**Specificity**
Height	0.969 (0.943–0.995)	≥169.00 cm	0.91	0.92
Lung—left bronchus	0.8 (0.713–0.887)	≥0.90 cm	0.85	0.71
Mediastinal—left bronchus	0.788 (0.701–0.875)	≥1.03 cm	0.94	0.5
Lung—tracheal transverse	0.757 (0.663–0.852)	≥1.82 cm	0.74	0.76

κ: Cohen’s kappa; ROC: receiver operating characteristic; AUC: area under the curve; CI: Confidence Interval.

**Table 5:** Cross-validated prediction of the tube size used by demographic and combined models.

Model	Exact Match (%)	Within One Size (%)	R^2^ (95% CI)	AUC (≥39 Fr) (95% CI)
Clinical estimate	85.7	100	0.787 (0.668–0.867)	0.918 (0.913–0.988)
Height + sex	80.7	100	0.745 (0.659–0.810)	0.972 (0.946–0.994)
Height + sex + lung-window left bronchus	80.7	100	0.748 (0.656–0.813)	0.973 (0.949–0.993)
Height + sex + weight + lung-window left bronchus	80.7	100	0.745 (0.656–0.812)	0.969 (0.951–0.993)
Lung-window left bronchus alone	54.3	96.4	0.237 (0.085–0.351)	0.788 (0.699–0.879)

AUC: area under the curve.

## Data Availability

The data presented in this study are available on request from the corresponding author and provided after ethics committee approval from the Gulhane Training and Research Hospital. The data are not publicly available as per the “Personal Data Protection Authority” of Turkey and due to hospital policy requiring ethics approval.

## Supplementary Materials

The following supporting information can be downloaded at: https://www.mdpi.com/article/10.3390/jcm15145415/s1, Table S1: Tube sizes used and lung-window left main bronchus diameter, Table S2: Ordinal modeling of double-lumen tube size and discrimination of adjacent sizes.

## Abbreviations

The following abbreviations are used in this manuscript:

3D	Three-dimensional
AP	Anteroposterior
AUC	Area under the curve
BMI	Body mass index
COPD	Chronic obstructive pulmonary disease
CT	Computed tomography
DLT	Double-lumen tube
Fr	French gauge
HRCT	High-resolution computed tomography
HU	Hounsfield unit
IQR	Interquartile range
MICS-CABG	Minimally invasive coronary artery bypass surgery
ROC	Receiver operating characteristic
SD	Standard deviation
SPSS	Statistical Package for the Social Sciences
