# Supplementary material for: Role of High-Resolution Computed Tomography in Double-Lumen Tube Selection for Patients Undergoing Minimally Invasive Coronary Bypass
Source: J Clin Med. 2026 Jul 10;15(14):5415. doi: 10.3390/jcm15145415 (PMC13410308; doi:10.3390/jcm15145415)
Supplement: Supplementary file 1 [file jcm-15-05415-s001.zip › jcm-4407818-supplementary.pdf]

Suppl. Table S1: Tube sizes used and lung-window left main bronchus diameter

Height band (cm)	Patients (n)	Tube sizes used, n	Lung-window left bronchus (cm, mean ± SD)	Within-band ρ (bronchus vs tube)
<160	5	35 Fr: 5	0.82 ± 0.03	— (single size)
160–164	17	35 Fr: 2, 37 Fr: 14, 39 Fr: 1	0.83 ± 0.19	0.44 (p = 0.080)
165–169	24	35 Fr: 1, 37 Fr: 13, 39 Fr: 10	0.96 ± 0.17	0.39 (p = 0.058)
170–174	41	35 Fr: 1, 37 Fr: 2, 39 Fr: 36, 41 Fr: 2	1.05 ± 0.17	0.24 (p = 0.126)
175–179	29	39 Fr: 22, 41 Fr: 7	1.02 ± 0.14	0.42 (p = 0.024)
180–184	19	39 Fr: 4, 41 Fr: 15	1.11 ± 0.16	-0.27 (p = 0.260)
≥185	5	41 Fr: 5	1.14 ± 0.10	— (single size)

Within-band ρ is the Spearman correlation between the lung-window left main bronchus diameter and the tube size used, computed where ≥ 2 sizes were used and n ≥ 5. Fr: French gauge, SD: standard deviation.

Suppl. Table S2: Ordinal modeling of double-lumen tube size and discrimination of adjacent sizes.

**Proportional-odds ordinal logistic model of the tube size used (n = 140)**	
**Predictor**	**Odds ratio (95% CI)**
Height (per 1 cm)	1.68 (1.47–1.92)
Height (per 5 cm)	13.3 (6.8–25.9)
Male sex (vs female)	18.2 (1.8–182.6)

**Discrimination of adjacent tube sizes (area under the ROC curve, 95% CI)**			
**Adjacent pair**	**n**	**Height**	**Lung-window left bronchus**
35 vs 37 Fr	38	0.818 (0.595–1.000)	0.716 (0.551–0.882)
37 vs 39 Fr	102	0.954 (0.914–0.995)	0.728 (0.613–0.843)
39 vs 41 Fr	102	0.925 (0.861–0.988)	0.658 (0.540–0.776)

Ordinal model: proportional-odds cumulative logistic regression; an odds ratio above 1 indicates that a higher value of the predictor is associated with a larger tube size. The proportional-odds assumption was satisfied for height but weaker for sex, as nearly all recipients of the largest tube were men.
Adjacent-size discrimination: for each pair, the area under the ROC curve is the probability that a patient receiving the larger size had the higher value; 95% CIs by the DeLong method. CI, confidence interval.
